# Supplementary material for: Identification of photoperiod-induced specific miRNAs in the adrenal glands of Sunite sheep (Ovis aries)
Source: Front Vet Sci. 2022 Jul 22;9:888207. doi: 10.3389/fvets.2022.888207 (PMC9354845; doi:10.3389/fvets.2022.888207)
Supplement: Supplementary file 1 [file Data_Sheet_1.ZIP › Supplementary file/miRDeep2 file.zip!/miRDeep2 file/Novel_Structure/SPLP42A1/Novel_419.pdf]

Provisional ID	: NC_019478.2_27414
Score total	: 0
Score for star read(s)	: -1.3
Score for read counts	: 0
Score for mfe	: -0.3
Score for randfold	: 1.6
Score for cons. seed	:
Total read count	: 32
Mature read count	: 32
Loop read count	: 0
Star read count	: 0

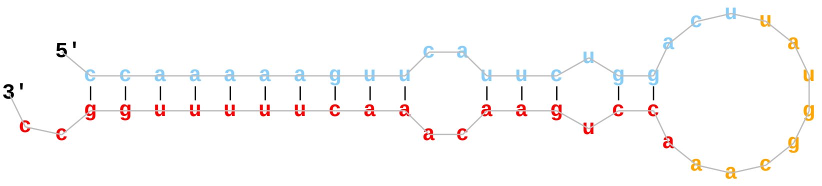

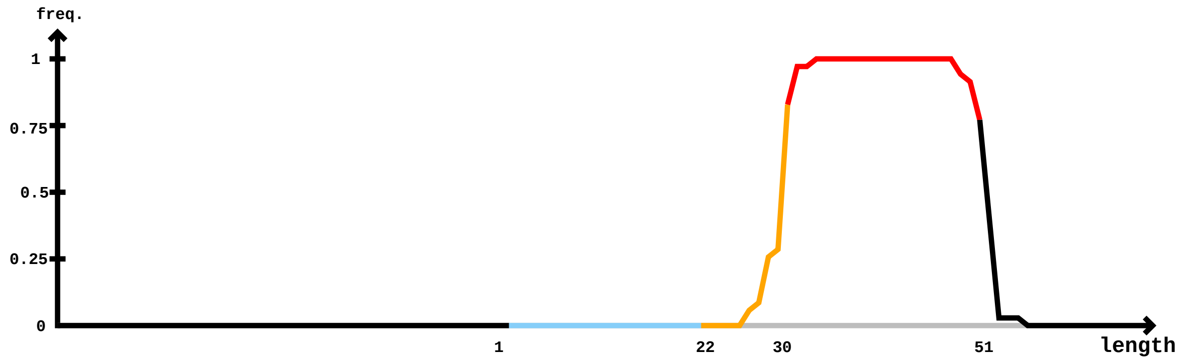

Star Mature

```
5'- uguugugagaaaaccaccaccuguuuuguaaaaguaucaggauggccaaaaaguucauucuggacuuauggcaaaccugaacaaacuuuuuggccaacucaaaaguuuuuug  -3'   exp
    .......(((((((.....((((..............)))).(((((((((((((..(((.((............)).)))..)))))))))))))........))))))).  reads   mm   sample
    ......................................................................gcaaaccugaacaaacuuuuug....................  1       0    seq
    ......................................................................gAaaaccugaacaaacuuuuug....................  1       1    seq
    .......................................................................Aaaaccugaacaaacuuuuugg...................  1       1    seq
    ........................................................................aaaccugaacaaacuuuuuggc..................  3       0    seq
    ........................................................................aaaAcugaacaaacuuuuuggc..................  1       1    seq
    ........................................................................aaaUcugaacaaacuuuuuggcc.................  1       1    seq
    ........................................................................aaaccugaacaaacuuuuuggcA.................  1       1    seq
    .........................................................................aaUcugaacaaacuuuuuggc..................  1       1    seq
    ..........................................................................accugaacaaacuuuuuggcA.................  1       1    seq
    ..........................................................................aUcugaacaaacuuuuuggcc.................  18      1    seq
    ...........................................................................ccugaacaaacuuuuuggcU.................  1       1    seq
    ...........................................................................Ucugaacaaacuuuuuggcca................  4       1    seq
    .............................................................................ugaacGaacuuuuuggccaacu.............  1       1    seq
```
